# Investigation of Antioxidant and Antimicrobial Activities of Different Extracts of* Auricularia* and* Termitomyces* Species of Mushrooms

**DOI:** 10.1155/2019/7357048

**Published:** 2019-07-24

**Authors:** Gebreselema Gebreyohannes, Andrew Nyerere, Christine Bii, Desta B. Sbhatu

**Affiliations:** ^1^Department of Biological and Chemical Engineering, Mekelle Institute of Technology, Mekelle University, Ethiopia; ^2^Molecular Biology and Biotechnology, Pan African University, Institute for Basic Sciences, Technology, and Innovation, Nairobi, Kenya; ^3^Department of Medical Microbiology, College of Health Sciences, Jomo Kenyatta University of Agriculture and Technology, Nairobi, Kenya; ^4^Center for Microbiology Research, Kenya Medical Research Institute, Nairobi, Kenya

## Abstract

Mushrooms produce a variety of bioactive compounds that are known to have a potential source of antioxidant and antimicrobial properties. Natural antioxidants can protect against free radicals without any side effects. The purpose of this study was to evaluate the antioxidant and antimicrobial activities of* Auricularia* and* Termitomyces* extracts. Specimens of* Auricularia* and* Termitomyces* spp. were collected from Kakamega National Reserve Forest in Kenya. Specimens were identified, extracted, and screened for their antioxidant and antimicrobial activities using stable free radical DPPH and colorimetric bioassay methods, respectively. The antimicrobial activity of the extracts was tested against* Escherichia coli, Klebsiella pneumoniae*,* Pseudomonas aeruginosa*,* Staphylococcus aureus, *MRSA,* Candida albicans*, and* Candida parapsilosis*. The maximum scavenging activity of hot water extract of* Auricularia* spp. was observed at 70.4% with the IC_50_ value of 40 *μ*g/mL. Of the three extracts of* Termitomyces* spp., 70% ethanol extract has shown the highest scavenging activity (63%) with the IC_50_ value of 50 *μ*g/mL. Chloroform and hot water extracts of* Auricularia* have shown statistically significantly different antifungal activities against* C. parapsilosis* (*df *= 2, F = 22.49,* p *≤ 0.05). Of all the organisms,* S. aureus* was highly susceptible to 70% ethanol and hot water extracts of* Termitomyces* spp. with minimum inhibitory concentration values of 0.67±0.29 mg/mL.* S. aureus* and* E. coli* were the most susceptible and resistant bacteria to the hot water extract, respectively. In conclusion, the extracts of* Auricularia* spp. and* Termitomyces* spp. have shown promising antimicrobial and antioxidant activities.

## 1. Introduction

In the last few years, several antimicrobial and antioxidant compounds were discovered from the Fungi Kingdom [1, 2]. Mushrooms possess enormous biologically active secondary metabolites for different applications [3–7]. Moreover, they have a wide range of secondary metabolites of high therapeutic values such as antioxidant, diabetes, antiviral, antithrombotic, anti-inflammatory, and antitumor activities [8–10]. It has been reported that a total of 316 mushroom species extracts have shown antimicrobial activities against Gram-positive and Gram-negative bacteria [11–13].

Though most mushroom species, if not all, are immensely rich in bioactive compounds, they are largely untapped resource of useful natural compounds [14, 15]. Evaluation of antioxidant and antimicrobial activities of mushroom extracts is currently the focus of the research [16]. However, the information about the effects of* Auricularia and Termitomyces *spp. extracts on antioxidant and antimicrobial activities is scarce. Extended knowledge of these mushroom species extracts may have remarkable importance to develop novel compounds that have antioxidant and antimicrobial benefits to be used as functional additives into our food regime to prevent damage caused by oxidation and pathogenic organisms. Therefore, this study examined* in vitro* antioxidant and antimicrobial activities of chloroform, 70% ethanol, and hot water extracts of the fruiting body of* Auricularia and Termitomyces *spp.

## 2. Materials and Methods

### 2.1. Collection and Identification of Mushroom Specimens

**Figure 1:**
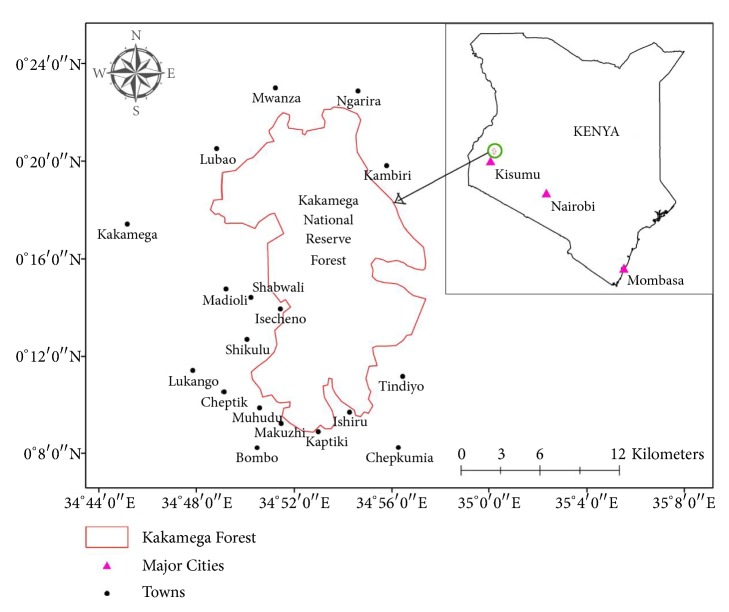
Map of the Kakamega National Reserve Forest.

Specimens of* Auricularia* and* Termitomyces* indigenous wild mushrooms were collected from Kakamega National Reserve Forest in Kenya in the months of March and May 2017 (Figure 1). The specimens were identified using molecular analysis and taxonomical keys by comparing their morphological characteristics with related literature [17–21].

### 2.2. Extraction Process of the Mushroom Specimens

**Figure 2:**
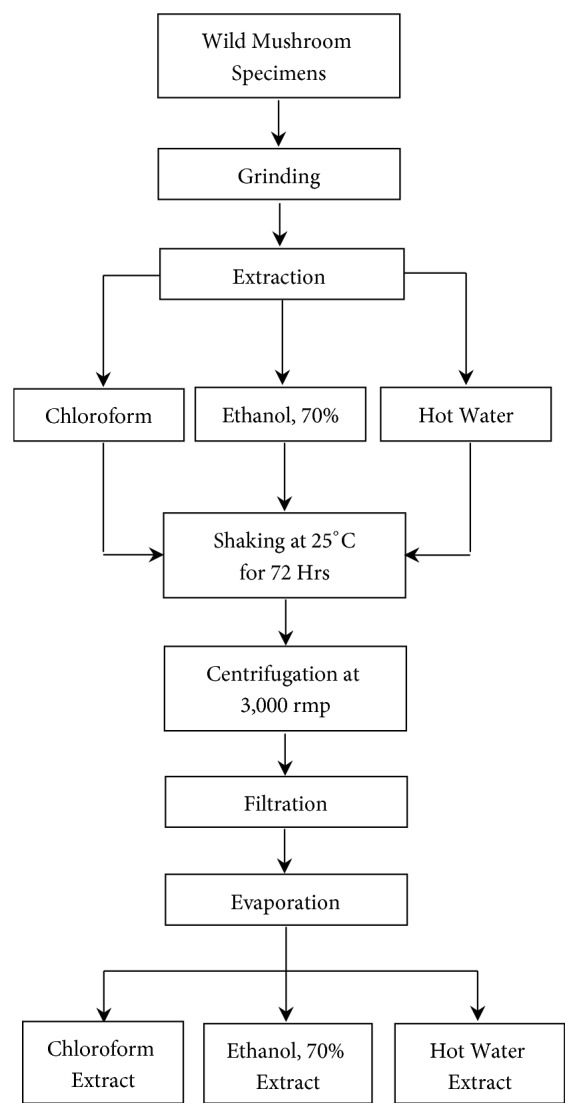
Extraction process of mushroom specimens.

Mushroom extraction was carried out using chloroform, 70% ethanol, and distilled hot water [22]. Dry mushroom specimens were pulverized and 100 g of powder was mixed with 0.5 L of chloroform, 70% ethanol, and distilled hot water (60°C) solvents in an Erlenmeyer flask at 25°C (Figure 2). The content was shaken using an incubator shaker at 150 rpm for 72 h. The extracts were centrifuged at 3000 rpm for 15 min, filtered with Whatman No. 1 filter paper, and dried by a rotary evaporator at 50°C. The extracts were kept in –80°C deep freezer and freeze dried. Finally, they were stored in the 4°C refrigerator in an amber colored bottle for further analyses.

### 2.3. Antioxidant Activities of the Extracts of* Auricularia* and* Termitomyces* spp.

The antioxidant activity of the chloroform, 70% ethanol, and hot water extracts of* Auricularia* spp. and* Termitomyces* spp. was determined using the stable free radical DPPH with little modification from the previous method [23, 24]. About 4 mL of 400 *μ*M of 2, 2-Diphenyl-1-picrylhydrazyl (DPPH) was dissolved in chloroform, ethanol, and distilled water. Different concentrations of the extracts (10, 20, 30, 40, 50, 60, and 70 *μ*g/mL) were prepared in a test tube and 1 mL of 400 *μ*M of DPPH was added. The mixture was shaken vigorously and left at room temperature for 60 min in a dark place until stable absorption values were obtained. Briefly, 1 mL of chloroform, 70% ethanol, distilled water, and DPPH were prepared without extracts as negative controls. The color change from purple to yellow was observed in terms of absorbance using a spectrophotometer at 517 nm. Different concentrations of ascorbic acid (10, 20, 30, 40, 50, 60, and 70 *μ*g/mL) were prepared and used as a standard antioxidant (positive control). Radical scavenging activity was expressed as the inhibition percentage of free radical by the extracts and was calculated using the formula: % RSA = [(ADPPH-AS)/ADPPH] x100, where AS is the absorbance of the solution (extract +DPPH) and ADPPH is the absorbance of the DPPH solution. The assay was carried out in triplicate and the results were expressed as mean values ± standard deviations. The extract concentration providing 50% inhibition (IC_50_) was calculated from the graph of RSA percentage against extract concentration [25]. Likewise, the standard antioxidant (ascorbic acid) was calculated as a percentage of DPPH discoloration using the equation: % RSA = [(ADPPH-AAA)/ADPPH] X100, where AAA is the absorbance of ascorbic acid.

### 2.4. Preparation of Test Organisms

Six bacterial species,* E. coli *(CI)*, K. pneumoniae* (ATCC 13883),* P. aeruginosa* (CI and ATCC 27853),* S. aureus *(ATCC 25923), and MRSA (ATCC 33591) as well as two yeast species,* C. albicans* (CI) and* C. parapsilosis* (ATCC 90018), were used as test organisms. The bacterial species were grown in 5 mL of Mueller-Hinton broth at 37°C for 12–16 h, while yeast species in 5 mL Sabouraud dextrose broth at 30°C for 24 h. The inoculum size of each test organism was adjusted to a concentration of 1.5 × 10^8^ CFU/mL by comparing with 0.5 McFarland standards.

### 2.5. Determination of Antimicrobial Activities of Mushroom Extracts

The AMA of mushroom extracts was determined using 2, 3, 5-triphenyl-tetrazolium chloride (TTC) microtiter plate bioassay. Stock solutions of mushroom extracts were prepared by dissolving 20 mg/mL of each extract in DMSO. Moreover, Mueller-Hinton broth was prepared. First, 100 *μ*L of MHB was poured into each of the 96 (8 rows by 12 columns) wells of the microtiter plate. Then, the 100 *μ*L of the extract was added to Well #1 in Row A and two-fold serial dilution was done through Well #9. The Wells #10, #11, and #12 were left with no mushroom extracts and were designated as “positive control,” “quality control,” and “negative control,” respectively. Wells #1 through #9 in the remaining rows (rows B through H) were filled with a mixture of MHB and mushroom extracts with similar concentrations. Finally, the last three wells were left for positive, quality, and negative controls, respectively.

With the exception, Well #11 in each row, the contents of Wells #1 through #12 were inoculated with 100 *μ*L of test organism in triplicate. Chloramphenicol and Clotrimazole drugs (0.1*μ*g/mL) were served as a positive control against bacterial and yeast species, respectively. The microtiter plate was incubated at 37°C and 30°C for 24 h for bacteria and yeast, respectively. After 24 h of incubation, 50 *μ*L of 0.2mg/mL TTC was added into the wells of the microtiter plate and then incubated for 30 min to 3 h at 37°C and 30°C for bacteria and yeast species, respectively. The MBC and MFC were determined by taking a loopful of inoculum from each well of the microtiter plate and streaking it on Mueller-Hinton and Sabouraud dextrose agar plates for bacteria and yeast species, respectively. The bacteria and yeast-streaked plates were incubated for 24 h at 37°C and 30°C, respectively.

### 2.6. Data Collection and Analyses

Data sources are antioxidant activities, MIC, MBC, and MFC. The MIC data were collected by a color change, while MBC and MFC data by growth inhibition of test organisms. Concentrations that result in color change and/or growth inhibition were recorded as data. Then, the data were subjected to ANOVA using the SPSS Version 24.0 statistical software. Inferences were made at* a priori* significance level of* p* ≤ 0.05.

## 3. Results

### 3.1. Antioxidant Activities of* Auricularia* Extracts

**Figure 3:**
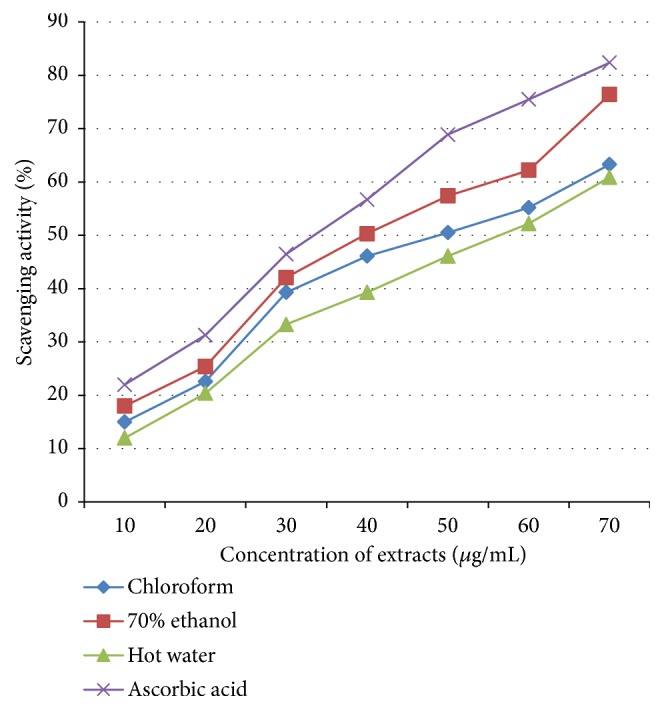
Free radical scavenging activities of* Auricularia *extracts.

During the free radical scavenging assay, it was observed that the extracts showed the dose-dependent percentage of scavenging activities. All extracts of* Auricularia* spp. revealed strong antioxidant activities. The antioxidant activity was expressed by the inhibitory concentration (IC_50_) value, which is the amount of extract needed to decrease 50% of the initial concentration of the free radical. The extracts were found to be potent antioxidants in the concentration range of 40-60 *μ*g/mL to scavenge and neutralize 50% of the free radical. The free radical scavenging activity was increased with increased concentrations of the extracts (Figure 3). Even though all extracts of* Auricularia* spp. revealed promising antioxidant activities, they were not as good as the standard antioxidant (ascorbic acid) (Figure 3).

### 3.2. Antioxidant Activities of* Termitomyces* Extracts

**Figure 4:**
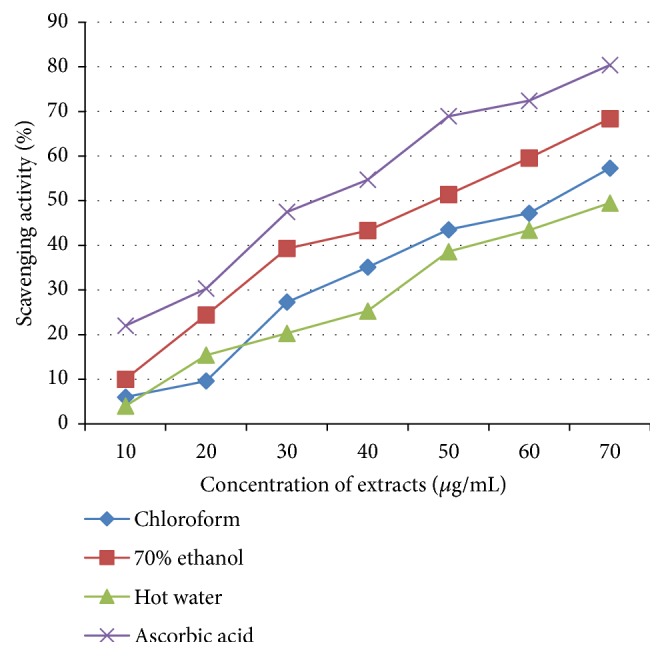
Free radical scavenging activity of* Termitomyces* extracts.

All extracts of* Termitomyces* spp. showed considerable free radical scavenging activities at a limited range of concentrations (10-70 *μ*g/mL). The results of the extracts were evaluated and compared with a standard antioxidant (ascorbic acid). But, none of them have revealed better scavenging activity than the standard antioxidant (Figure 4). Of the extracts, 70% of ethanol extract exhibited the highest scavenging activity (63%) by demonstrating inhibition concentration (IC_50_) value at 50 *μ*g/mL. However, the IC_50_ values of chloroform and hot water extracts were observed at 65 *μ*g/mL and 70 *μ*g/mL, respectively.

### 3.3. Antimicrobial Activity of* Auricularia* Extracts

Chloroform, ethanol, and HWE of* Auricularia* spp. were subjected to antimicrobial screening and the results were promising (Table 1).* E. coli*,* K. pneumoniae* (ATCC 13883),* C. parapsilosis* (ATCC 90018), and* S. aureus* (ATCC 25923) were observed to be the most resistant organisms to the AMA of the chloroform extract of* Auricularia* species. Ethanol and HWE have shown the strongest AMA against* S. aureus* (ATCC 25923). Ethanol extract has resulted in limited AMA against* E. coli* and* P. aeruginosa* (CI). On the other hand, all extracts have resulted in the same AFA against* C. albicans *with chloroform and HWE showing statistically significantly different AMA against* E. coli *(*df *= 2, F = 7,* p *≤ 0.05). All extracts have shown potent AMA against Gram-positive bacteria compared to Gram-negative bacteria and yeast (Table 1).

The MFC of chloroform, ethanol, and hot water extracts of* Auricularia* spp. were also analyzed and results are presented below (Table 1). HWE has resulted in the strongest MBC and MFC activities against* S. aureus* (ATCC 25923) and* C. parapsilosis* (ATCC 90018), respectively. Chloroform extract has resulted in statistically significantly different AMA against* E. coli*,* P. aeruginosa* (CI),* S. aureus *(ATCC 25923),* C. albicans, *and* C. parapsilosis* (ATCC 90018) only (*df* = 7, F = 2.29,* p *≤ 0.05). On the other hand, the chloroform and HWE have shown statistically significantly different AFA against* C. parapsilosis* (ATCC 90018) (*df *= 2, F = 22.49,* p *≤ 0.05). In conclusion, extracts of* Auricularia* spp. were generally less effective against* E. coli* (Gram-negative bacterium) as compared to* S. aureus *(Gram-positive bacterium).

### 3.4. Antimicrobial Activity of* Termitomyces* Extracts

Different extracts of* Termitomyces* spp. were also tested against clinically isolated human pathogens and standard strains. The minimum inhibitory values of hot water extracts against the test organisms ranged from 0.67±0.29 mg/mL to 1.00 mg/mL (Table 2). Of all test organisms,* S. aureus* (ATCC 25923) was highly susceptible to chloroform, ethanol, and hot water extracts with MIC values of 0.83 ± 0.29 mg/mL, 0.67 ± 0.29 mg/mL, and 0.67 ± 0.29 mg/mL, respectively. All extracts of* Termitomyces* spp. were tested for their AMA against* E. coli. *Chloroform and HWE have shown statistically significantly different growth inhibitory activities against* E. coli* (*df* = 2, F = 5.6,* p *≤ 0.05).

HWE was active against* S. aureus* (ATCC 25923),* K. pneumoniae* (ATCC 13883),* P. aeruginosa* (CI),* P. aeruginosa* (ATTC 27853), MRSA (ATCC 33591), and* C. albicans*.* S. aureus* (ATCC 25923) was found to be the most susceptible bacterium to HWE followed by* K. pneumoniae* (ATCC 13883), MRSA (ATCC 33591),* P. aeruginosa* (CI),* P. aeruginosa* (ATTC 27853), and* C. albicans*. HWE was found to be more potent against* C. albicans *(CI) than* C. parapsilosis* (ATCC 90018). On the other hand,* E. coli *(CI) was found to be the most resistant organism to HWE. These results showed that the HWE of* Termitomyces* spp. possesses AFA and ABA.

## 4. Discussion

In the current study, all extracts of* Auricularia* and* Termitomyces* spp. were found to be potent antioxidants in the concentration range of 40-60*μ*g/mL and 10-70*μ*g/mL, respectively. However, a previous study reported that the radical scavenging activity of mushroom extracts was shown at 2.11 mg/mL [26]. The possible reasons for the varied results of antioxidant activities between the present and previous study might be due to the different extraction conditions, and the difference in the presence of phenolic compounds and other secondary metabolites [27–29]. Many other studies also reported that phenolic compounds obtained from mushroom extracts have shown excellent antioxidant activities by scavenging free radicals [30–33].

*Auricularia* and* Termitomyces* spp. extracts have shown remarkable AMA against the test organisms. However, the chloroform, 70% ethanol, and HWE of* Auricularia* and* Termitomyces* extracts have exhibited varying degrees of AMA. The observed variations may be due to the presence of different bioactive substances and their mechanism of action against test pathogenic organisms. Moreover, the AMA difference among the extracts of the two mushrooms could be attributed to their difference in species and variations in the composition and concentrations of secondary metabolites within the extracts [34]. Another result obtained from the research done on the AMA of some local mushrooms against pathogenic isolates also suggested that the AMA of the extracts might have various potent bioactive compounds [12]. A study conducted on the AMA of extracts of* Trametes elegans* also reported that the differences in the AMA of the extracts have been mainly attributed to the ability of solvents to extract different bioactive components from the mushrooms [35, 36]. Similarly, Barros et al. [37] findings also verified that the AMA of the mushroom extracts were a result of the presence of various arrays of secondary metabolites.

The findings of the current study indicated that HWE of* Auricularia* and* Termitomyces* mushrooms have shown strong ABA against* S. aureus* compared to other test organisms. This might be associated with the synergistic effect and the broad-spectrum ABA of HWE. A study done by Cai et al. [38] also established that the HWE of* Auricularia auricula-judae* exhibited the greatest ABA against* S. aureus*. The observed disparity in the AMA of HWE against the test organisms may be correlated with its higher polarity and ability to extract powerful bioactive compounds. In corroborative to the present findings, previous studies also reported that polar solvents were found to be the most effective in extracting potent organic and inorganic mycochemicals [39]. The chemical nature of the bioactive compounds and the type and pH of the solvents can also have an influence on the AMA of the extracts [22]. Furthermore, the strong AMA of HWE against the microbes could be attributed to its ability to dissolve endogenous compounds [40].

On the other hand, the* Auricularia* and* Termitomyces* species chloroform and ethanol extracts revealed weaker AMA against test organisms. A previous study also reported that 70% of alcohol was not effective in extracting antimicrobial compounds from the fruiting bodies of* P. ostreatus,* which is related to the present findings [41]. The weak AMA of the chloroform and ethanol extracts might be the absence of bioactive compounds in the extracts or lost activities due to heat or chemical transformation into inactive products during extraction [12]. These deviations might also be associated with the difference in the contents and concentrations of the antimicrobial compounds present in the aqueous and organic solvent extracts [42, 43].

All extracts of* Auricularia* and* Termitomyces* species exhibited high AMA against Gram-positive bacteria compared to Gram-negative bacteria and yeast, which approves the previous results on the ABA of aqueous extracts of eight edible mushrooms [44]. It was proven that bioactive compounds obtained from the extracts of many mushroom spp. demonstrated higher antimicrobial activities against Gram-positive compared to Gram-negative bacteria [45]. The variation of AMA observed between the Gram-negative and Gram-positive bacteria might be attributed to the nature of genetic makeup of the tested organisms, structure, composition differences of the cell wall of the bacteria, mycochemical composition, and concentrations of the extracts, type, and nature of solvents used for extraction, and mechanisms of actions of bioactive compounds [22, 46–48].

All tested extracts have shown a marked difference in their AMA towards the clinical isolates and standard strains. The clinical bacteria and yeast isolates were found to be more resistant to the extracts than the standard strains. The main reason for the resistance of the clinical isolates might be directly linked to the indiscriminate exposure of the clinical isolates to various antimicrobial agents [48, 49]. According to Taiwo [50], several clinical isolates have effective antibiotic resistance mechanisms through the acquisition of resistant genes, production of enzymes (e.g., ß-lactamases), and efflux pumping of the drug out of the cell.

## 5. Conclusion

All extracts of* Auricularia* and* Termitomyces* mushrooms have revealed potent antioxidant activities. They have also shown promising antimicrobial activities against the tested organisms. Of all extracts, hot water extract of both mushroom species has shown strong antibacterial activity against* S. aureus. *However, further studies into isolation, structural elucidation, identification, and determination of the mechanisms of action of the bioactive compounds of the mushroom extracts responsible for the antioxidant and antimicrobial activities are compulsory.

## Figures and Tables

**Table 1:** Antimicrobial activity of *Auricularia* extracts against pathogenic organisms.

Tested organisms	Chloroform extract (mg/mL)			70% Ethanol Extract (mg/mL)			HWE (mg/mL)		
	MIC	MBC/MFC	+ve (*μ*g/mL)	MIC	MBC/MFC	+ve (*μ*g/mL)	MIC	MBC/MFC	+ve (*μ*g/mL)
*E. coli* (Clinical Isolate)	2.00±0.00^a^	2.00±0.00^i^	0.10±0.00^p^	1.33±0.58^p^	1.33±0.58^p^	0.10±0.00^p^	1.00±0.00^b^	1.17±0.76^**f**^	0.10±0.00^p^
*K. pneumoniae* (ATCC 13883)	1.33±0.58^p^	1.33±0.58^p^	0.10±0.00^p^	1.00±0.00^p^	1.33±0.58^p^	0.10±0.00^p^	0.83±0.29^p^	0.83±0.29^p^	0.10±0.00^p^
*P. aeruginosa* (Clinical Isolate)	2.00±0.00^p^	2.00±0.00^b^	0.10±0.00^p^	1.67±0.58^p^	1.00±0.00^p^	0.10±0.00^p^	1.33±0.58^p^	1.00±0.00^p^	0.10±0.00^p^
*P. aeruginosa* (ATTC 27853)	1.67±0.58^p^	1.67±0.58^p^	0.10±0.00^p^	1.33±0.58^p^	1.33±0.58^p^	0.10±0.00^p^	1.33±0.58^p^	0.83±0.29^p^	0.10±0.00^p^
MRSA (ATCC 33591)	1.00±0.00^p^	1.33±0.58^p^	0.10±0.00^p^	1.00±0.00^p^	1.00±0.00^p^	0.10±0.00^p^	1.00±0.00^p^	1.00±0.00^p^	0.10±0.00^p^
*S. aureus* (ATCC 25923)	1.00±0.00^p^	1.00±0.00^c^	0.10±0.00^p^	0.83±0.29^p^	0.83±0.29^p^	0.10±0.00^p^	0.83±0.29^p^	0.67±0.29^p^	0.10±0.00^p^
*C. albicans* (Clinical Isolate)	1.33±0.58^p^	2.00±0.00^d^	0.10±0.00^p^	1.33±0.58^p^	1.33±0.58^p^	0.10±0.00^p^	1.00±0.00^p^	1.33±0.58^p^	0.10±0.00^p^
*C. parapsilosis* (ATCC 90018)	1.33±0.58^p^	2.00±0.00^ef^	0.10±0.00^p^	1.00±0.00^p^	1.33±0.58^p^	0.10±0.00^p^	0.83±0.29^p^	1.00±0.00^g^	0.10±0.00^p^

HWE: hot water extract; MIC: minimum inhibitory concentrations; MBC/MFC: minimum bactericidal/fungicidal concentrations; +ve: positive control. Values are mean ± SD of three replicates. Values marked by different superscript letters within a column are statistically significantly different at *p *≤ 0.05. Values marked by different superscript letters within a row are statistically significantly different at *p *≤ 0.05.

**Table 2:** Antimicrobial activity of *Termitomyces* extracts against pathogenic organisms.

Tested organisms	Chloroform Extract (mg/mL)			70% Ethanol Extract (mg/mL)			HWE (mg/mL)		
	MIC	MBC/MFC	+ve (*μ*g/mL)	MIC	MBC/MFC	+ve (*μ*g/mL)	MIC	MBC/MFC	+ve (*μ*g/mL)
*E. coli* (clinical isolate)	1.67±0.58^a^	1.67±0.58^ab^	0.10±0.00^b^	1.00±0.00^ac^	1.50±0.87^bc^	0.10±0.00^cd^	0.83±0.29^a^	1.67±0.58^ee^	0.10±0.00^ff^
*K. pneumoniae* (ATCC 13883)	1.17±0.76^b^	1.67±0.58^ac^	0.10±0.00^c^	1.00±0.00^ad^	1.33±0.58^bd^	0.10±0.00^ce^	0.83±0.29^dc^	0.83±0.29^ea^	0.10±0.00^fa^
*P. aeruginosa* (clinical isolate)	1.67±0.58^c^	1.67±0.58^ad^	0.10±0.00^d^	1.33±0.58^af^	1.00±0.00^be^	0.10±0.00^cf^	1.00±0.00^dd^	0.83±0.29^eb^	0.10±0.00^fb^
*P. aeruginosa* (ATTC 27853)	1.33±0.58^d^	1.33±0.58^ae^	0.10±0.00^e^	1.00±0.00^ag^	1.00±0.00^bf^	0.10±0.00^cg^	0.83±0.29^de^	0.83±0.29^ec^	0.10±0.00^fc^
MRSA (ATCC 33591)	1.33±0.58^e^	1.00±0.00^af^	0.10±0.00^f^	0.83±0.29^ah^	1.00±0.00^bg^	0.10±0.00^ch^	0.83±0.29^df^	0.83±0.29^ed^	0.10±0.00^fd^
*S. aureus* (ATCC 25923)	0.83±0.29^f^	0.83±0.29^ag^	0.10±0.00^g^	0.67±0.29^ai^	0.67±0.29^bh^	0.10±0.00^ci^	0.67±0.29^dg^	0.50±0.00^ef^	0.10±0.00^fe^
*C. albicans* (clinical isolate)	1.67±0.58^g^	1.67±0.58^ah^	0.10±0.00^h^	1.00±0.00^aj^	1.00±0.00^bi^	0.10±0.00^cj^	0.83±0.29^dh^	0.83±0.29^eg^	0.10±0.00^fg^
*C. parapsilosis* (ATCC 90018)	1.67±0.58^h^	1.67±0.58^ai^	0.10±0.00^i^	1.00±0.00^ak^	1.33±0.58^bj^	0.10±0.00^ck^	0.83±0.29^di^	1.67±0.58^eh^	0.10±0.00^fh^

HWE: hot water extract; MIC: minimum inhibitory concentrations; MBC/MFC: minimum bactericidal/fungicidal concentrations; +ve: positive control. Values are mean ± SD of three replicates. Values marked by the same superscript letters within a column are statistically significantly different at *p *≤ 0.05. Values marked by the same superscript letters within a row are statistically significantly different at *p *≤ 0.05.

## Data Availability

The data used to support the findings of this study are included within the article. In any case, if the data used to support the findings of this study are required by anybody, they are available from the corresponding author upon request.

## Abbreviations

AMA: Antimicrobial activity
ABA: Antibacterial activity
AFA: Antifungal activity
MRSA: Meticillin resistant* Staphylococcus aureus*
CFU: Colony forming unit
CI: Clinical isolates
HWE: Hot water extract
MIC: Minimum inhibitory concentration
MFC: Minimum fungicidal concentration
MBC: Minimum bactericidal concentration
MHB: Mueller-Hinton broth
ANOVA: One-Way Analysis of Variance
DMSO: Dimethyl sulfoxide.
